# Flexible 3D Electrodes of Free-Standing TiN Nanotube Arrays Grown by Atomic Layer Deposition with a Ti Interlayer as an Adhesion Promoter

**DOI:** 10.3390/nano10030409

**Published:** 2020-02-26

**Authors:** Seokjung Yun, Sang-Joon Kim, Jaesung Youn, Hoon Kim, Jeongjae Ryu, Changdeuck Bae, Kwangsoo No, Seungbum Hong

**Affiliations:** 1 Department of Materials Science and Engineering, KAIST, Daejeon 305-701, Korea; best1017sj@kaist.ac.kr (S.Y.); yjs9465@gmail.com (J.Y.); huluddu@kaist.ac.kr (H.K.); meroojj@kaist.ac.kr (J.R.); 2 Center for Environment & Sustainable Resources, Korea Research Institute of Chemical Technology (KRICT), 141 Gajeong-ro, Daejeon 34114, Korea; sangjoon@krict.re.kr; 3 Department of Energy Science, Sungkyunkwan University, Suwon 440-746, Korea; changdeuck@skku.edu; 4 KAIST Institute for NanoCentury (KINC), KAIST, Daejeon 34141, Korea

**Keywords:** atomic layer deposition, flexible device, TiN nanotube, adhesion promoter, 3D electrode, nanomaterials

## Abstract

Nanostructured electrodes and their flexible integrated systems have great potential for many applications, including electrochemical energy storage, electrocatalysis and solid-state memory devices, given their ability to improve faradaic reaction sites by large surface area. Although many processing techniques have been employed to fabricate nanostructured electrodes onto flexible substrates, these present limitations in terms of achieving flexible electrodes with high mechanical stability. In this study, the adhesion, mechanical properties and flexibility of TiN nanotube arrays on a Pt substrate were improved using a Ti interlayer. Highly ordered and well-aligned TiN nanotube arrays were fabricated on a Pt substrate using a template-assisted method with an anodic aluminum oxide (AAO) template and atomic layer deposition (ALD) system. We show that with the use of a Ti interlayer between the TiN nanotube arrays and Pt substrate, the TiN nanotube arrays could perfectly attach to the Pt substrate without delamination and faceted phenomena. Furthermore, the I-V curve measurements confirmed that the electric contact between the TiN nanotube arrays and substrate for use as an electrode was excellent, and its flexibility was also good for use in flexible electronic devices. Future efforts will be directed toward the fabrication of embedded electrodes in flexible plastic substrates by employing the concepts demonstrated in this study.

## 1. Introduction

Nanostructured metals and semiconductors can offer synergetic effects by maximizing combined surface and bulk properties. Fabricating nanostructured metal electrodes is particularly challenging because wet-chemistry methods for metallic nanostructures are limited compared to their semiconducting oxide counterparts. Indeed, a wide range of oxide semiconductor nanostructures can be prepared by hydrothermal methods [1]. Recent reports highlight new functional oxides with nanostructures, including CoO_x_/graphene, V_2_O_5_, Zn_x_Co_1−x_O and Cu-Fe_3_O_4_, by means of combined preparation methodologies of chemical vapor deposition (CVD), electrochemical deposition and sol-gel [2,3,4,5]. However, metallic electrodes possessing three-dimensional (3D) nanostructures have rarely been investigated except for carbon nanotubes (CNTs) by CVD [6,7,8]. Metallic nanoelectrodes could serve as an ideal current collector in flexible integrated energy systems. An et al. introduced Zn-based supercapacitors with high energy-storage performance, outstanding mechanical flexibility and waterproof characteristics using carbon fiber-2D Zn flake electrodes [9]. Schnorr et al. and Zhu et al. reported various applications, such as field-effect transistors, sensors, transparent conductive films, organic light-emitting diodes, batteries and supercapacitors, using CNTs and graphene electrodes [10,11].

3D electrodes on flexible substrates are also key to the realization of flexible energy conversion and storage. At the nanoscale, metal electrodes in integrated circuits should possess superior mechanical, electrical and thermal properties. As such, conductive nitride, i.e., titanium nitride (TiN), is promising due to its good toughness, high electrical and thermal conductivity, hardness, melting point, and thermal and chemical stability [12,13,14,15,16,17,18]. Recently, TiN, as an electrode, has been adopted for ferroelectric devices, including hafnia-based ferroelectric memories [19,20]. Nanostructured TiN is also attractive for application in electrochemical energy storage even in corrosive environments [13,21] and photovoltaic devices with unusual absorption characteristics [22]. TiN nanostructures have been fabricated either by radio frequency (RF) magnetron sputtering [23], electrochemical processes [24] and hydrothermal processes [25]. Although above mentioned various processing methods can fabricate TiN nanostructures, atomic layer deposition (ALD) has strength in fabricating conformally grown films via saturated and sequential surface reactions. In addition, ALD can form a homogeneous thin coating layer of different materials with surface modification [26]. ALD allows for excellent deposition conformality and step coverage with high aspect-ratio, even in nanoporous and 3-dimensional structures [27,28].

Template-directed fabrication is one of the most common techniques in the formation of vertically aligned nanotube arrays and could allow for precise control over the physical dimensions, such as diameter, length and interpore distance [29,30,31,32]. However, nanomaterials such as nanotube arrays created by templated fabrication need additional supporting substrates. Although alumina templates (anodic aluminum oxide; AAO) grown directly on a Si substrate have been proposed [33], uniformly removing the thin barrier layers of AAO remains elusive. Another method is to deposit a metal layer on the top surface of nanotube (NT)/AAO composites; in this case, adhesion between the nanotubes (NTs) and supporting substrate is essential because the mechanical stability and electrical contact determine the resulting performance and further applications, including flexible integrated energy systems.

In this study, a great improvement in the adhesion between TiN NT arrays and the flexible substrates using a Ti interlayer was reported. Previously, highly ordered and vertically aligned TiN NT arrays were reported by using template-assisted ALD [34]. The TiN NTs on Pt layers likely delaminated as a result of the poor adhesion properties under the wet etching process to remove the template or bending conditions [35]. When employing very thin Ti as an interlayer, however, the TiN NT electrodes were perfectly glued to flexible substrates [36,37,38]. The delamination phenomena were not observed even upon bending tests at high curvature (radius of curvature, −2 mm). Moreover, the resulting secure and uniform contacts from conductive atomic force microscope (C-AFM) analysis also exhibited excellent local transport properties.

## 2. Materials and Methods

**Figure 1:**
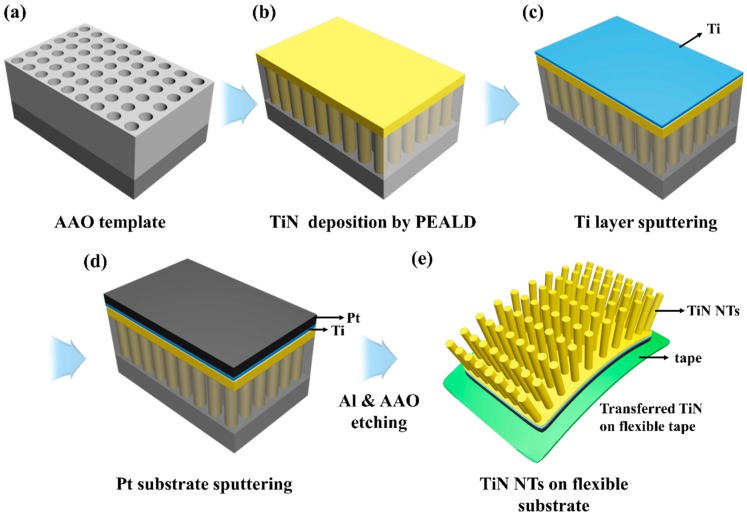
(**a**) A schematic illustration of the preparation of flexible TiN nanotube arrays. (**a**) anodized aluminum oxide (AAO) template, (**b**) deposition of TiN on the AAO by plasma enhanced atomic layer deposition (PEALD), (**c**) Ti, (**d**) Pt layer sputtered onto the top of deposited TiN, (**e**) TiN nanotubes (NTs) on a flexible substrate after removing the AAO template.

Figure 1 depicts a schematic illustration of the process flow to fabricate flexible TiN NTs. Fabrication began by anodizing aluminum foils (99.99% purity, 0.5 mm thickness, Alfa Aesar), and porous alumina templates were obtained through well-known two-step anodization procedures. Al was first electropolished under a constant voltage of 20 V at room temperature for 1 min in a solution of perchloric acid (ACS reagent, 70%; Sigma-Aldrich, St. Louis, MO, USA) and ethanol (volume ratio of 1:4). The first anodization was performed under a constant voltage of 40 V at 3 °C for 10 h in a solution with 0.3 M oxalic acid (≥99%; Sigma-Aldrich) as an electrolyte. The resulting alumina layers were removed via wet-chemical etching in a solution of phosphoric acid (6 wt% in H_2_O; Sigma-Aldrich) and chromic acid (1.8 wt% in H_2_O; Sigma-Aldrich) at 50 °C overnight. Upon the etching process, hexagonally patterned periodic concaves were obtained; these acted as self-assembled imprints for nucleation in the second anodization step. The second anodization step was performed under identical conditions as the first anodization step, except for the anodization time. To promote pore widening, the as-formed template was immersed in a 0.2 M phosphoric acid solution for an appropriate time. The porous alumina (anodized aluminum oxide; AAO) used in this work had hexagonally packed nanopores typically −60 nm in diameter and −300 nm in length (Figure 1a).

TiN NTs were formed on AAO membranes as a template at a temperature of 150 °C via plasma-enhanced ALD (DAEKI HI-TECH Co., Ltd., Daejeon, Korea). Tetrakis-dimethyl-amino-titanium (TDMAT; 99.999% purity; DNF Co. Ltd., Daejeon, Korea) and N_2_ plasma were employed as precursors, and Ar was commonly used as a carrier and purge gas. One full cycle consisted of a 2 s exposure to the Ti source, 10 s Ar purge, 20 s N_2_ plasma and 10 s Ar purge. The Ti and/or Pt layers were subsequently deposited on the previously grown TiN-deposited membranes by sputtering prior to attachment to flexible substrates (Figure 1b). To mechanically and electrically support the TiN NTs, Ti and Pt layers were deposited onto the TiN deposited side by general DC sputtering (Figure 1c,d).

To obtain free-standing TiN NT arrays, the AAO templates were removed by the following procedures. The aluminum foil was dissolved in a saturated solution of mercury chloride, and the alumina layer was then removed through treatment with a solution of phosphoric acid (6 wt%) and chromic acid (1.8 wt%) at 50 °C (Figure 1e). The morphology of TiN NT arrays was characterized by using field emission scanning electron microscopy (FESEM; Hitachi S-4800 SEM, Tokyo, Japan) and transmission electron microscopy (TEM; Tecnai G2 F30, FEI Company, Hillsboro, OR, USA). The structures were determined using X-ray diffraction (XRD; D/MAX-RC diffractometer, Rigaku, Tokyo, Japan) with Cu Kα irradiation (λ = 1.5418 Å). X-ray photoelectron spectroscopy (XPS) data were executed using K-alpha X-ray photoelectron spectroscopy (Thermo VG Scientific, Waltham, MA, USA) with an Al K-alpha X-ray source operating at 12 kV at a chamber pressure below 1 × 10^−8^ torr. Topography and transport properties were investigated using a conductive atomic force microscope (C-AFM; SPA-300HV, Seiko, Tokyo, Japan) with Pt-coated conductive AFM tips (Nanoworld, EFM-50, −2.8 N/m). The flexibility was tested using a cyclic bending test machine.

## 3. Results & Discussion

**Figure 2:**
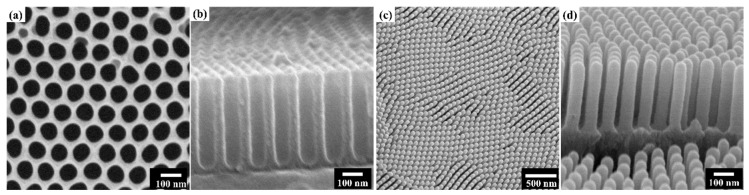
SEM images of (**a**) top view and (**b**) cross-section view of the AAO template, (**c**) top view and (**d**) cross-section view of well-aligned TiN NTs.

Figure 2a,b shows the fabricated AAO template, which was prepared electrochemically featuring hexagonally well-arranged pores with a diameter of −60 nm (Figure 2a) and a length of −300 nm (Figure 2b). Vertically ordered and well-aligned TiN NTs are shown in Figure 2c,d. TiN NTs were successfully formed after the removal of the AAO template without any bundling or clustering. The TiN NT tops were closed, the insides were hollow, and the diameter and length of NTs were consistent with the pore size and depth of the AAO template, respectively.

**Figure 3:**
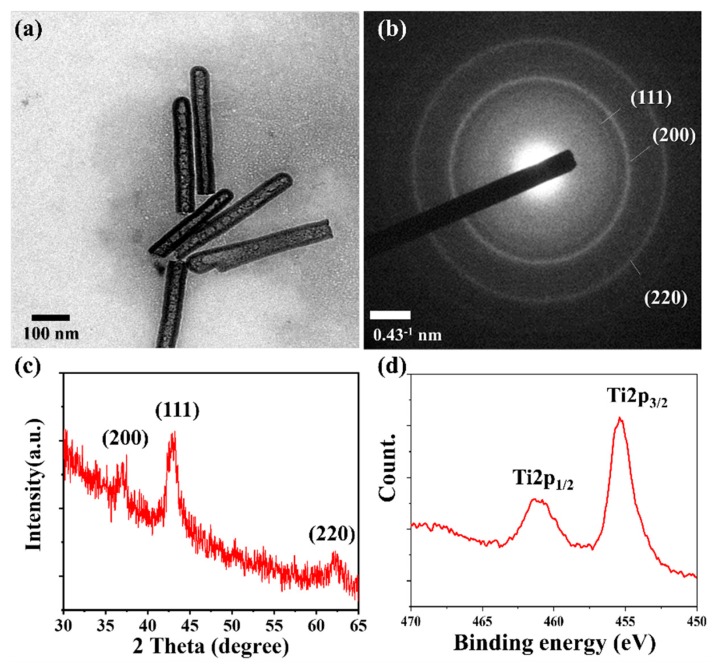
Fabricated TiN NTs, (**a**) TEM image of NTs, (**b**) SAED pattern, (**c**) XRD patterns, and (**d**) XPS peaks of Ti 2p for TiN.

To examine the detailed morphology and microstructure of TiN NTs, TEM analysis was carried out. The TEM image of the TiN NTs is shown in Figure 3a, and the NTs obviously exhibit a tubular shape. The wall thickness of the TiN nanotube was observed to be −15 nm. As shown in Figure 3b, the selected area electron diffraction (SAED) patterns confirmed the polycrystalline characteristics of TiN with lattice planes of (111), (200) and (220). To further study the crystal structures of the fabricated TiN NTs, XRD analysis was performed (Figure 3c). The peaks related to the directions of a face-centered cubic TiN lattice were clearly observed in the XRD patterns, which were well-matched with the SAED pattern. The chemical state of the TiN NTs was verified by X-ray photoelectron spectrometry (XPS) analysis. The Ti 2p peaks in the XPS spectrum revealed two characteristic peaks at 455 eV for 2p_3/2_ and 461 eV for 2p_1/2_ with an energy gap of 6 eV between the 2p_3/2_ and 2p_1/2_ peaks (Figure 3b) [39].

**Figure 4:**
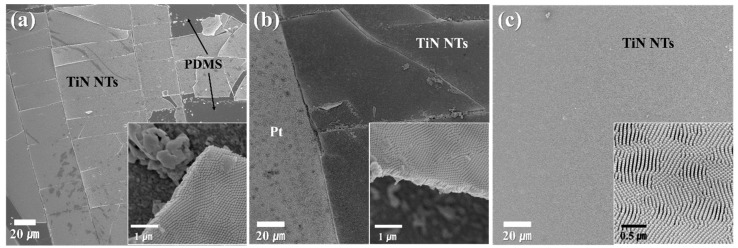
Surface morphologies of TiN nanotube arrays transferred to different flexible substrates: (**a**) PDMS, and (**b**) Pt layers, (**c**) Ti and Pt laminated substrate; insets correspond to magnified images.

Our TiN NT arrays were transferred to different types of flexible substrates, such as polydimethylsiloxane (PDMS), Pt, and Ti/Pt, as shown in Figure 4a–c, respectively. All the cases displayed that the resultant NT arrays peeled off, delaminated and faceted from the substrates due to poor adhesion properties. On the basis of the lateral dimension of the delaminated array pieces, the Pt layer exhibited high mechanical bonding properties compared with the PDMS substrate. Despite the good adhesion to Pt, the phenomena of peeling-off and cracks in the NT arrays make flexible device application difficult even with possibly small mechanical shocks. However, improvement in the bonding properties of TiN NT arrays with Pt was achieved by inserting Ti as an interlayer, as shown in Figure 4c. This is because Ti forms a strong bonding with both Pt substrate and TiN NTs [40,41,42]. The NT electrodes were attached to the substrate without any delamination and cracking, indicative of the presence of a good interlayer of Ti between TiN and Pt.

**Figure 5:**
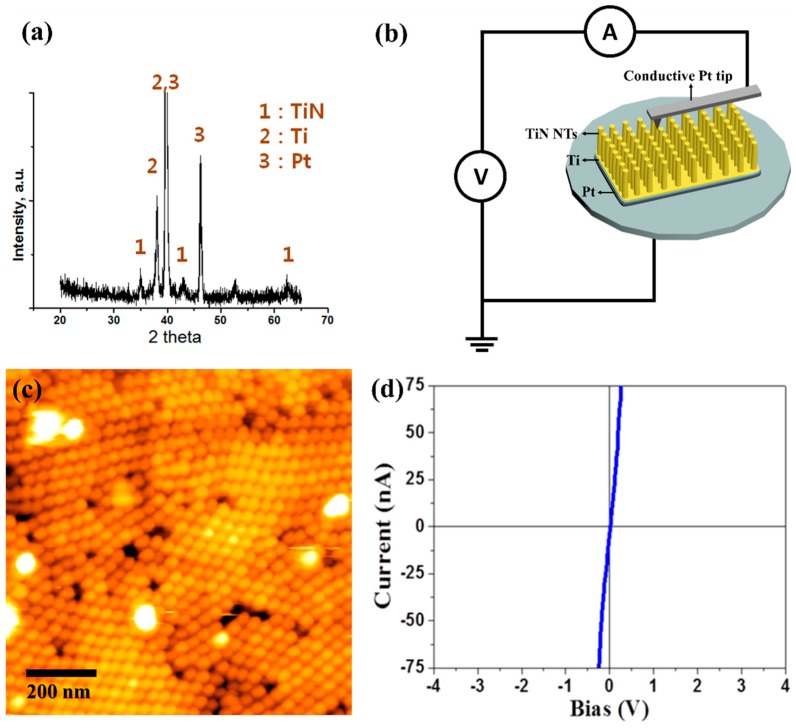
Analysis of TiN NTs/ Ti / Pt samples (**a**) XRD, (**b**) schematic of C-AFM setup, (**c**) AFM height image, and (**d**) local I-V curve by C-AFM.

We confirmed that the Ti interlayer was deposited between TiN NTs and Pt substrates by X-ray diffraction (Figure 5a). The high and sharp peak intensities, which belong to Ti and Pt, indicate their good crystallinities. The stable and uniform electrical junctions between TiN and Pt were confirmed using conductive atomic force microscopy (C-AFM) [43,44,45]. The schematic of C-AFM setup, morphologies and I-V curves of the TiN NT arrays on the Ti/Pt substrate are given in Figure 5b–d. For C-AFM, the scan area was 1 μm × 1 μm and the I-V curve was acquired by sweeping the tip bias from +5 to −5 V. The AFM height image shows highly ordered and well-aligned NT arrays (Figure 5c). The electric circuit of the C-AFM setup is schematically described in Figure 5b, where the tip is in contact with TiN NT that is connected in series with TiN NT/Ti/Pt interface. Therefore, if any of the components show rectifying behavior (e.g., Schottky contact), then the overall I-V from the C-AFM setup will show non-linear behavior [46,47]. The transport measurement at a local region in Figure 5d exhibits a linear behavior, indicative of the ohmic contact between our tube arrays and flexible substrate.

**Figure 6:**
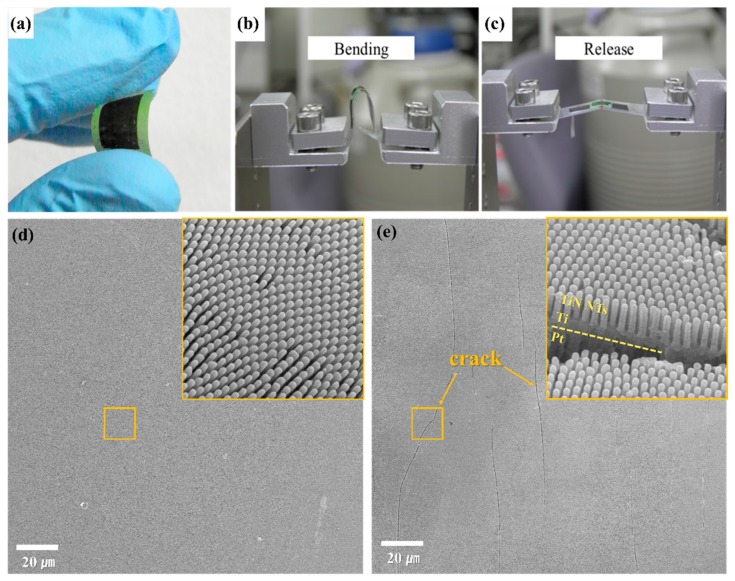
(**a**) Photograph of flexible TiN NT arrays. Cyclic bending (**b**) and releasing (**c**) tests of our array devices. SEM images of the TiN NT arrays upon cyclic bending tests after (**d**) 1000 and (**e**) 3000 cycles.

The capability of bending is related to successful and reliable operation in flexible integrated devices. The flexibility of the fabricated TiN NT Ti/Pt/flexible substrate (tape) was investigated using the cyclic bending and releasing method at high curvature (radius of curvature, −2 mm), as shown in Figure 6a–c. This method has been previously reported in detail [48,49]. Figure 6d,e shows the resulting surface morphologies after 1000 and 3000 bending cycles, respectively. No noticeable cracks or delamination were detected upon operation after 1000 cycles (Figure 6d). After 3000 bending cycles (Figure 6e), however, it was observed that small cracks along the bending direction at the center of the sample were formed. Although small cracks were observed after 3000 cycles of bending and release, delamination and faceted phenomena were not observed, implying that this method has great potential for application in flexible systems as a 3D electrode.

## 4. Conclusions

In conclusion, we investigated the formation of TiN NT arrays on flexible substrates by template-directed ALD and presented a comparative study on the utility of Ti as an adhesion promoter in the present flexible electrodes. The enhanced adhesion of the TiN NTs/Ti/Pt substrate was demonstrated by local transport measurements and bending tests at high curvature (−2 mm in a radius of curvature). Although cracks were observed after 3000 cycles of bending and release, severe delamination was not observed. The present strategy provides a new class of nanostructured 3D electrodes to overcome critical mechanical stability, thus providing a great potential platform for application in a flexible integrated device.
